# Rationale and design of a randomized controlled trial of varenicline directly observed therapy delivered in methadone clinics

**DOI:** 10.1186/1940-0640-9-9

**Published:** 2014-06-13

**Authors:** Shadi Nahvi, Kate S Segal, Alain H Litwin, Julia H Arnsten

**Affiliations:** 1 Department of Medicine, Albert Einstein College of Medicine and Montefiore Medical Center, 111 East 210th Street, 10467 Bronx, NY, USA; 2 Department of Psychiatry & Behavioral Sciences, Albert Einstein College of Medicine, 111 East 210th Street, 10467 Bronx, NY, USA; 3 Department of Epidemiology & Population Health, Albert Einstein College of Medicine, 10467 Bronx, NY, USA

**Keywords:** Adherence, Smoking cessation, Varenicline, Directly observed therapy, DOT, Methadone, Opioid-related disorders

## Abstract

**Background:**

Tobacco cessation medication adherence is one of the few factors shown to improve smoking cessation rates among methadone-maintained smokers, but interventions to improve adherence to smoking cessation medications have not yet been tested among methadone treatment patients. Methadone clinic-based, directly observed therapy (DOT) programs for HIV and tuberculosis improve adherence and clinical outcomes, but have not been evaluated for smoking cessation. We describe a randomized controlled trial to evaluate whether a methadone clinic-based, directly observed varenicline therapy program increases adherence and tobacco abstinence among opioid-dependent drug users receiving methadone treatment.

**Methods/Design:**

We plan to enroll 100 methadone-maintained smokers and randomize them to directly observed varenicline dispensed with daily methadone doses or treatment as usual (self-administered varenicline) for 12 weeks. Our outcome measures are: 1) pill count adherence and 2) carbon monoxide-verified tobacco abstinence. We will assess differences in adherence and abstinence between the two treatment arms using repeated measures models.

**Discussion:**

This trial will allow for rigorous evaluation of the efficacy of methadone clinic-based, directly observed varenicline for improving adherence and smoking cessation outcomes. This detailed description of trial methodology can serve as a template for the development of future DOT programs and can guide protocols for studies among opioid-dependent smokers receiving methadone treatment.

**Trial Registration:**

clinicaltrials.gov NCT01378858

## Background

Methadone maintenance treatment patients have a disproportionately high prevalence of tobacco use and suffer high rates of tobacco-related disease and mortality [1-6]. Cessation approaches evaluated to date among opioid-dependent smokers, including nicotine replacement therapy, bupropion, or varenicline in combination with behavioral therapy, have not been effective over control conditions [7-11]. Varenicline’s demonstrated efficacy may not be generalizable to methadone-maintained smokers because of poor adherence, which is highly prevalent among drug users [11-15]. Though adherence to smoking cessation treatment is a critical determinant of successful cessation [16-23] and adherence is one of the few factors shown to increase cessation among methadone-maintained smokers [12,24,25], interventions to improve adherence to smoking cessation pharmacotherapy have not yet been tested in this group.

Administration of directly observed therapy (DOT) for tuberculosis (TB) and HIV regimens at opioid agonist treatment programs has been shown to improve adherence and clinical outcomes among methadone maintenance patients [26-30]. Methadone maintenance programs provide an ideal setting for DOT administration. Regulatory requirements mandate that patients attend methadone programs up to 6 times weekly, minimizing barriers to medication adherence, and providing a platform for treatment of other diseases. Because of the limited treatment course and low pill burden of smoking cessation treatment, it may be even more feasible to implement DOT for smoking cessation treatment than for TB or HIV treatment. To our knowledge, no studies to date have evaluated DOT for smoking cessation.

We thus designed a randomized trial of modified, directly observed versus self-administered varenicline therapy in methadone clinics to evaluate the efficacy of modified DOT (mDOT) for improving varenicline adherence and smoking cessation among methadone-maintained smokers, and to test whether drug use and psychiatric symptoms moderate the effects of mDOT on adherence.

## Methods/Design

This is a randomized pilot trial of modified directly observed versus self-administered varenicline therapy among 100 methadone-maintained smokers. We will evaluate the efficacy of mDOT varenicline for promoting smoking cessation and medication adherence with research visits at baseline and at 1, 2, 3, 6, 9, 12, and 24 weeks. We will also evaluate the moderating effects of illicit drug use and psychiatric symptoms on mDOT adherence effects.

### Study setting

Participants will be recruited from the Einstein Division of Substance Abuse (DoSA) methadone clinics, which offer integrated substance abuse and medical treatment to 3200 patients in three clinical sites in the Bronx, New York. Established in 1968, DoSA is a clinical, research, and educational division of the Einstein Department of Psychiatry. Each clinic offers comprehensive medical services, integrating general and HIV-related medical and gynecologic services with co-located substance abuse treatment.

### Participants

Our goal is to enroll subjects representative of methadone-maintained smokers, while considering varenicline precautions. Eligible persons are: 18 years or older; smoking 5 or more cigarettes per day; interested in quitting smoking with a plan to quit in ≤ 6 months (ladder of change score 6–8); enrolled in methadone treatment for at least 3 months, receiving methadone in the clinic 3, 4, 5, or 6 times per week, without three or more clinic absences in the prior 2 weeks; English-speaking; able to provide informed consent; not pregnant, breastfeeding, or trying to conceive; and have not taken varenicline in the past 30 days. Unstable liver, cardiac, pulmonary, renal, or infectious diseases are exclusionary. Psychiatric exclusion criteria include current major depressive or manic episode, current psychotic disorder, past-year suicide attempt or psychiatric hospitalization, or current suicidal ideation with plan or intent.

Subjects will be recruited by research assistants in methadone clinic waiting areas, as well as by word-of-mouth, posted fliers, and clinic counseling and medical staff referral. All subjects will sign written informed consent. We will conduct a brief screening interview, then abstract laboratory and treatment data from clinic charts to further assess eligibility. Eligibility will be confirmed only after the study physician completes a clinical and structured psychiatric interview using the Mini-International Neuropsychiatric Interview 6.0.0 (M.I.N.I.) and Columbia Suicide Severity Rating Scale, and conducts a physical examination and urine pregnancy test (if applicable). Interviews will be completed in private offices in the methadone clinics.

### Interventions

Subjects will receive varenicline for 12 weeks, using standard dosing: 0.5 mg once daily for 3 days, then 0.5 mg twice daily for 4 days, followed by 1 mg twice daily for 11 weeks. Subjects will be randomly assigned to receive varenicline via: 1) treatment as usual (TAU) or 2) modified directly observed therapy (mDOT). Most subjects will have public insurance that should cover the cost of varenicline; for those who do not, varenicline will be purchased from a local pharmacy using research funds.

### DOT Intervention

**Figure 1:**
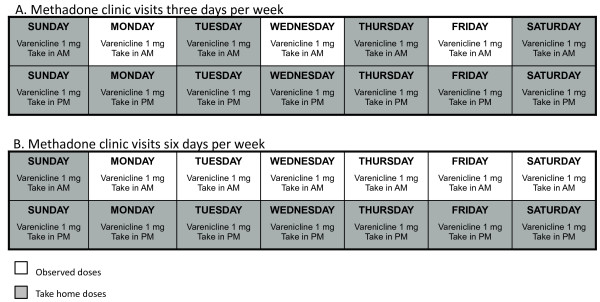
**Example mDOT varenicline pill trays. A**. Methadone clinic visits 3 days per week. **B**. Methadone clinic visits 6 days per week. This figure illustrates mDOT varenicline pill box labels indicating observed and take-home varenicline doses, according to subjects’ methadone clinic schedule.

Subjects in the mDOT arm will receive: 1) directly observed varenicline doses at the same time as they receive a methadone dose at their methadone program, between 3 and 6 times per week; and 2) take-home doses packaged in individual pill boxes for self-administration on days off and evenings (Figure 1). In addition, methadone clinic nurses will assess side effects using a standard script and will refer subjects to on-site medical providers for treatment if side effects are reported.

We describe the intervention as ‘modified directly observed therapy’ because varenicline ingestion will be observed at the methadone window 3–6 days per week based on the subject’s methadone clinic schedule. Furthermore, varenicline is a twice-daily medication, and only one of two daily varenicline doses can be observed. For example, if a subject receives methadone at his/her program Monday through Saturday and a take-home dose of methadone on Sunday, s/he would receive observed varenicline at the same time s/he receives methadone Monday through Saturday (for a total of six observed doses), and would get pill boxes for evening and Sunday varenicline doses (eight unobserved doses weekly). Subjects will be instructed to return the pill boxes for unobserved doses at their next clinic visit, whether or not they have taken the pills.

To minimize nursing burden, trays of pill boxes will be prepared by research assistants, indicating varenicline dosing and dates of administration, and color-coded labels will indicate whether the dose is directly observed or self-administered (for days off and evenings). Each tray will hold seven removable pill boxes, with compartments for morning and evening varenicline doses. The study physician will call in varenicline prescriptions to a single, designated, community pharmacy that delivers medications to the DoSA central pharmacy. Empty, labeled trays also will be delivered to the central DoSA pharmacist, who will fill pill boxes with varenicline for each subject. Filled trays will be delivered from the central pharmacy to the methadone clinics every 2 weeks, along with scheduled methadone deliveries.

### TAU Control condition

Subjects in the TAU arm will pick up prescribed varenicline from a local pharmacy of their choosing and self-administer all varenicline doses (once daily for 3 days, then twice daily).

### Counseling and medication education

All subjects in both groups will receive a single session of brief, physician, smoking cessation counseling based on the Public Health Service 5As framework (ask, assess, advise, assist, arrange follow-up) [31]. Brief advice has been shown to increase the likelihood of successful smoking cessation [31], is associated with outcomes comparable to tailored motivational counseling among methadone-maintained smokers [8], and is easily implemented in complex clinical settings. Subjects also will receive a brochure promoting medication adherence, as well as verbal and written instructions on varenicline administration and management of anticipated side effects. If subjects report persistent or severe treatment-emergent symptoms over the intervention period, they will meet with the study physician for assessment and brief counseling on symptom management.

### Randomization

Subjects will be randomized a 1:1 ratio in variable size blocks of 2–8 via central, computer-generated randomization. Randomization will be stratified by clinical site and HIV status. We will randomize in blocks to ensure comparison groups of approximately equal size. To ensure concealment of allocation, a centrally-located data manager will generate the allocation sequence and store the sequence in a password-protected file. After the study physician confirms a subject’s eligibility, she will call the data manager, who will assign the subject to a treatment group. Since the intervention is not blinded, we will vary block size to prevent anticipation of treatment arm assignment.

### Visit schedule and measures

Research visits will be scheduled at baseline and at weeks 1, 2, 3, 6, 9, 12, and 24 (Table 1). We will collect survey data using Audio Computer-Assisted Self-Interview (ACASI), which has been shown to improve reporting of stigmatized behaviors. Subjects will be reimbursed up to $110 for completing all research assessments. Retention will be enhanced by flagging subjects for research visits at the methadone clinic reception desk, tracking contact information of subjects and their close contacts at each study visit, reimbursement for completion of assessments, and on-site data collection at subjects’ clinics.

**Table 1:** Timing of study measures

	**Baseline/Start Rx**						**End of treatment**	
**Week**		1	2	3	6	9	12	24
**Study measures**								
Sociodemographic characteristics	X							
Tobacco use behavior, FTND^a^	X			X	X	X	X	X
Smoking endpoints^b^				X	X	X	X	X
Medication adherence		X	X	X	X	X	X	
Mini-International Neuropsych. Interview	X			X	X	X	X	X
Modified scale for suicidal ideation	X			X	X	X	X	
Brief symptom Inventory	X			X	X	X	X	X
Medication adverse events		X	X	X	X	X	X	
Alcohol and other drug use	X			X	X	X	X	X
Urine toxicology tests	X			X	X		X	
Medication adverse events		X	X	X	X	X	X	
**Subject reimbursement**								
$ Amount^c^	$15	$10	$10	$15	$15	$15	$15	$15

^a^Fagerström Test for Nicotine Dependence.

^b^Carbon monoxide-verified abstinence, number of cigarettes smoked per day, Fagerström Test for Nicotine Dependence score, quit attempts lasting ≥ 24 hours.

^c^To ensure pill count completion, TAU subjects will be given partial reimbursement ($5-$10 dollars per visit) for research assessments and $5 for completion of pill counts at each follow-up visit during the intervention period.

### Adherence measures

Our primary outcome is adherence (as a continuous measure) by pill count. Pill counts are an objective measure that have been shown to correlate with electronic monitors [32]. The amount of medication taken during the 12-week treatment course will be expressed as a percentage of the target dose. These are established measures used in other DOT trials in our setting [29,33]. Self-report is the most practical adherence measure for clinical use, and it has been shown to correlate with clinically relevant outcomes in several studies [34-37]. Though self-report is subject to recall and reporting bias and may overestimate adherence [32,36,38,39], we will use self-report as a second measure of adherence because it is easy to administer and is generalizable. Because electronic monitors (using medication event monitoring systems [MEMs]) cannot be used in the mDOT arm, we have chosen not to use MEMs.

### Tobacco use measures

Our primary tobacco use outcome is biochemically validated 7-day point prevalence abstinence at each follow-up visit, with abstinence defined as including both self-reported smoking abstinence and carbon monoxide (CO) < 8 p.p.m. (Micro Smokerlyzer®, Bedfont Scientific) [40]. Self-reported tobacco abstinence will be measured at all visits by asking participants, “Have you smoked at least part of a cigarette in the past 7 days, even a puff?” Our secondary tobacco outcome measures include: 1) number of cigarettes smoked per day; 2) Fagerström Test for Nicotine Dependence score; 3) quit attempts lasting ≥ 24 hours; and 4) durability, measured by CO-verified tobacco abstinence at week 24.

### Potential moderating variables: psychologic symptoms

We will assess incident psychologic symptoms at weeks 3, 6, 9, and 12 using: 1) M.I.N.I. [41], which is a short, structured, diagnostic interview for DSM-IV Axis I disorders; 2) the Columbia Suicide Severity Rating Scale, a structured interview that assesses suicidal ideation, plans, intent, and behavior [42]; and 3) the Brief Symptom Inventory (BSI [43]), a measure of general psychological symptom status that can be monitored over time.

### Potential moderating variables: Alcohol and drug use

We will assess alcohol and drug use at baseline and at weeks 3, 6, 9, and 12 via: 1) the Addiction Severity Index, Lite version [44]; 2) the Alcohol Use Disorders Identification Test [45]; and 3) urine toxicology tests.

### Medication adverse effects

Adverse events will be evaluated at each follow-up visit during the intervention period, using both a structured questionnaire that assesses the presence of specific symptoms that have been reported among varenicline subjects in published clinical trials, and an open-ended review of treatment-emergent symptoms, defined as symptoms that emerged or increased in intensity following the start of study medication.

### Major hypotheses and analytic plans

We will review and summarize data using descriptive summaries and graphical analyses to ensure recorded values are within appropriate ranges and to check for outliers and abnormal values. Interim analyses will not guide recruitment decisions.

### Varenicline adherence

We hypothesize that adherence in the mDOT arm will be higher than in the TAU arm throughout the intervention period. We will apply a mixed effects linear model to test the significance of the mDOT effect on the repeatedly measured pill count adherence outcome at weeks 1, 2, 3, 6, 9, and 12. This model accounts for within-subject longitudinal outcome correlation by taking the subject-level intercept as random, and it is robust in the context of missing data. The model will also include variables not equally distributed between the study arms at baseline, as well as psychiatric symptoms and substance abuse. All analyses will use an intent-to-treat approach.

### Tobacco use

We hypothesize subjects in the mDOT arm will have greater CO-verified, 7-day point prevalence abstinence throughout the intervention period; reductions in cigarettes/day and Fagerström Test for Nicotine Dependence scores; and ≥ 24-hour quit attempts compared to subjects receiving self-administered varenicline. We will compare the proportion of participants who achieve CO-verified tobacco abstinence at all follow-up timepoints in the two study arms using mixed-effects models. In addition, we will compare secondary tobacco use outcomes between study arms using chi-square or Wilcoxon rank sum tests, and explore differences in CO-verified abstinence at 24 weeks using Fisher exact tests. All analyses will use an intent-to-treat approach. In addition, we will apply mixed effects models to test the significance of varenicline adherence on smoking cessation across the post-baseline assessment time points.

### Moderating effects of drug use and psychiatric symptoms

We hypothesize that ongoing drug use and psychiatric symptoms will negatively moderate adherence effects. The dependent variable will be varenicline adherence. Potential moderating variables include cocaine, heroin, benzodiazepine, or marijuana use (defined as a binary variable based on either self-reported use or positive urine toxicology result); hazardous alcohol use (defined as AUDIT score ≥ 8 for men and ≥ 4 for women); incident major depressive, manic, or psychotic episodes; suicidal ideation with plan or intent; and symptoms of psychologic distress (BSI Global Severity Index T score ≥ 63). We will test moderators using a treatment group x moderating variable interaction term, and set an alpha significance level of 0.15 for interaction analyses.

### Sample size and power considerations

Among methadone-maintained smokers receiving a nicotine patch in a clinical trial, overall adherence over 90 days was 44.1 percent [12]. Assuming a 13-percent difference in adherence between the DOT and TAU groups as seen in weeks 2–12 of a methadone clinic-based antiretroviral DOT trial [30], the hypothesized adherence effect would be 57 percent in the mDOT arm and 44 percent in the TAU arm. We estimate sample sizes N per group required to detect this effect at a two-sided α = 0.05 as N per group = 11, 14, and 17 for intraclass correlation (ICC) = 0.1, 0.2, and 0.3, respectively.

Given that the efficacy of NRT and bupropion in methadone maintenance patients has been half of that observed in clinical trials of these medications in the general population [7,8,15], we estimate that abstinence rates at 12 weeks in the TAU varenicline arm would be 22 percent. We estimate that increased adherence would double the odds of cessation [13,17,18,46], and thus the abstinence rates at 12 weeks in the DOT arm would be 44 percent. We estimate sample sizes required to detect the hypothesized intervention effect (22% and 44% on a binary scale) at a two-sided alpha = 0.05 as N per group = 29, 33, 38 for within-subject ICC = 0.1, 0.2, and 0.3, respectively. Estimating an approximately 25 percent attrition rate at 12 weeks, we plan to recruit 100 participants.

### Dissemination policy

We will disseminate study findings at national scientific meetings and in published manuscripts. On request, we will also share study protocols and de-identified study data with investigators for research purposes. No study participants will be individually identified in any published or shared data.

### Study design considerations

#### Optimizing pill count adherence measurement

For mDOT arm subjects, varenicline dosing will be evaluated by directly counting unconsumed varenicline pills remaining behind the methadone window, as well as those returned to nurses in pill boxes. Unobserved doses in unreturned pill boxes will be counted as nonadherence. Each day, nurses also will complete a pre-printed calendar documenting whether doses were dispensed, refused, or if the subject missed clinic.

Since a 4-week supply of 1 mg varenicline tablets can be dispensed in bottles (containing 56 tablets) or in four weekly blister packs (each containing 14 tablets), TAU group subjects will be asked to bring in the full month’s supply of medication to each study visit for pill counting. For TAU arm subjects, research assistants will count pills remaining in varenicline bottles or blister packs at weeks 1, 2, 3, 6, 9, and 12. Given that TAU subjects obtain a 4-week supply of medication at a time from community pharmacies, and that pill-count adherence would be challenging to interpret if no pills were remaining, we designed the research visit schedule to assess pill count adherence separately from the every-4-week medication dispensing intervals. To ensure pill count completion, TAU group subjects will be given partial reimbursement ($5-$10 dollars per visit) for completion of research assessments and $5 for completion of pill counts at each follow-up visit during the intervention period.

### Protection of study subjects

#### Confidentiality and research protections

The trial was approved by the Einstein Committee on Clinical Investigations. All subjects will complete an informed consent process and provide written consent. We also obtained a Certificate of Confidentiality from the National Institute on Drug Abuse to protect against disclosure of research information in federal, state, or local civil, criminal, or administrative proceedings. We established a Data Safety and Monitoring Committee (DSMC), composed of two senior clinical investigators with expertise in clinical trials. We will report trial progress to the DSMC quarterly, along with incident psychiatric illness, serious or unanticipated adverse events, pregnancies, and protocol deviations, and we will meet formally twice a year.

#### Addressing varenicline risk potential

Case reports of behavior change, agitation, depression, and suicidality among patients taking varenicline led to an FDA boxed warning in 2009 [47]. Despite a growing body of research demonstrating the safety of varenicline among patients with mental illness and substance use disorders [48-56], we will take multiple precautions to address varenicline’s psychiatric risk potential.

Prior to enrollment, information regarding potential psychiatric risks will be discussed with subjects during the informed consent process. During screening, a physician will assess potential subjects for current psychiatric illness or suicidal ideation using structured instruments. Individuals with psychiatric symptoms not receiving psychiatric care will be referred to a mental health center.

Subjects will be assessed for incident psychiatric symptoms throughout the intervention period. At brief visits (weeks 1 and 2), and weeks 3, 6, 9 and 12, the interviewer will ask open-ended questions about symptoms that have emerged or increased in intensity since the prior visit. At week 3, 6, 9, and 12 follow-up visits, psychiatric symptoms will be assessed via structured instruments. Prior to concluding the research visit, the research assistant will review flags programmed into the computerized survey record for symptoms consistent with incident major depressive or manic episode, psychotic disorder, or suicidal ideation and will refer subjects to outpatient or emergency treatment according to detailed study protocols.

If subjects meet criteria for current major depressive or manic episode, psychotic disorder, or suicidal ideation with plan or intent, varenicline will be discontinued. To facilitate medication discontinuation among TAU group subjects who fill varenicline prescriptions at community pharmacies of their choosing, prescriptions will not be given directly to study subjects; rather, the study physician will call in all prescriptions and track the pharmacies used. If DOT group subjects meet criteria for medication discontinuation, the study physician will notify the study pharmacy and the methadone clinic nurses.

## Discussion

This study represents the first randomized controlled trial of directly observed smoking cessation treatment in a methadone program. Our trial draws on prior work demonstrating the feasibility and efficacy of directly observed HIV and TB treatment [26-30] and extends this research to evaluate smoking cessation medication. Outcomes from this trial will contribute to knowledge about whether varenicline DOT is efficacious at promoting adherence and smoking cessation among methadone maintenance patients.

Multiple studies suggest that smoking cessation medication adherence is an important determinant of cessation success [16-21], including among methadone-maintained smokers [12,24,25]. In a retrospective cohort study in which methadone maintenance patients were prescribed varenicline during routine clinical care, varenicline treatment duration was significantly associated with smoking cessation [25]. In two large smoking cessation trials among methadone maintenance patients, adherence to nicotine patch treatment was also shown to be associated with improved smoking cessation outcomes [12,24]. Methadone-maintained smokers had fewer cigarettes per day and a 7.1x increased odds of abstinence on days in which they used patches compared to days they did not [12]. This supports the importance of developing and evaluating interventions to promote varenicline adherence.

Our trial has several strengths. Participants will be randomly assigned to receive DOT or self-administered varenicline. We will use objective measures of both adherence and smoking cessation. The core components of our DOT program will not require additional funding over existing clinic operating budgets. We will not provide salary support for nurses, and medications will be paid for by subjects’ existing insurance plans. We will supply pill boxes used for DOT, and we will pay the DoSA central pharmacist a stipend for filling the pill boxes.

This study also has limitations. Reasons for medication nonadherence are multifaceted. While a DOT strategy cannot address many potential adherence barriers, DOT interventions have demonstrated efficacy for improving adherence and clinical outcomes in TB and HIV. Generalizability to opioid-dependent persons outside of drug treatment may be limited. Nonetheless, over 200,000 people are enrolled in methadone programs in the US, over 80 percent of methadone maintenance patients smoke, and a significant number of methadone programs offer linked primary care services, including TB and HIV care. Furthermore, if proven effective, a DOT model could be used in other inpatient and outpatient substance abuse treatment settings. Finally, our modest sample size may not allow us to definitively evaluate moderating variables or to compare tobacco abstinence rates between the mDOT and TAU groups for smaller effect sizes. When we designed this study, there were no published studies on varenicline effects among methadone-maintained smokers. We have since completed a placebo-controlled trial of varenicline without DOT, in which 10.5 percent of varenicline-treated subjects were abstinent [57]. In another trial of varenicline, only 4 percent of methadone-maintained smokers achieved abstinence at the end of treatment [11].

Despite these potential limitations, we feel that our design will allow for a controlled evaluation of intervention effects, and our sample size will be adequate to assess adherence in both arms and to inform effect size estimation for future studies of smoking cessation interventions among methadone maintenance patients. Even if the cessation effect of this intervention is small, the potential health impact may be large, given the high prevalence of tobacco use and tobacco-related illness in substance abuse treatment patients.

This study has the potential to improve smoking cessation treatment outcomes and to inform the dissemination of smoking cessation services among substance abuse treatment patients. This, in turn, may reduce the disproportionate prevalence and associated disease burden of tobacco use in this difficult-to-treat group.

## Abbreviations

DOT: Directly observed therapy; mDOT: Modified directly observed therapy; TAU: Treatment as usual; DoSA: Division of Substance Abuse (Albert Einstein College of Medicine).

## Competing interests

The authors declare that they have no competing interests.

## Authors’ contributions

SN and JA designed the study, KS and AL made substantial contributions to study design, and SN wrote the first draft of the manuscript. All authors contributed to manuscript preparation and have read and approved the final manuscript.
